# Supplementary material for: Relationship between ferroptosis and mitophagy in acute lung injury: a mini-review
Source: PeerJ. 2024 Sep 10;12:e18062. doi: 10.7717/peerj.18062 (PMC11397134; doi:10.7717/peerj.18062)
Supplement: Supplemental Information 1 [file peerj-12-18062-s001.doc]

Dear Editors and Reviewers:

All authors, including myself, would like to extend our sincerest gratitude to the editors for reviewing our manuscript. We would like to provide detailed reasons for adding a new author, Liling Zhu. The detailed reasons are as follows.

1. Liling Zhu began participating in the revision of our article on June 11, 2024, after receiving feedback from the editor and reviewers.

2. Liling Zhu reviewed and revised the Introduction and Conclusions based on the reviewers' comments.

3. Liling Zhu added content on the relationship between ferroptosis and mitophagy in "3. Ferroptosis and Mitophagy in Acute Lung Injury" per the reviewers' suggestions and created Table 2, Figure 2, and Figure 3.

4. Liling Zhu reviewed and polished our expressions in sections such as the Abstract and "Ferroptosis and Mitophagy in Acute Lung Injury."

5. All current co-authors have agreed to the changes by replying to the confirmation email.

6. In our updated tracked changes manuscript, the content resulting from Liling Zhu's involvement has been indicated using the comment function.

We would like to express our gratitude once again. The above details provide our reasons for adding a new author (Liling Zhu). We sincerely hope that you will agree to the inclusion of this new author (Liling Zhu), and we confirm that this is our final list of authors. If you have any queries, please don’t hesitate to contact me at the address below.

Thank you and best regards.

Yours sincerely,

Yunhua Cheng   E-mail: chengyunhua97@163.com
